# Criterion validity of the 10 personality aspects for performance in South Africa

**DOI:** 10.4102/ajopa.v6i0.129

**Published:** 2024-04-16

**Authors:** Xander van Lill, Cobi Hayes

**Affiliations:** 1 Department of Industrial Psychology and People Management, College of Business and Economics, University of Johannesburg, Johannesburg, South Africa; 2 Department of Product and Research, JVR Africa Group, Johannesburg, South Africa; 3 Derivco, Durban, South Africa

**Keywords:** personality, 10 aspects, work performance, criterion validity, dominance analysis

## Abstract

Recent evidence suggests that personality could play just as important a role in the prediction of important work-related outcomes as cognitive ability. However, it might be meaningful to adjust the level at which personality is measured and reported, depending on the narrowness of the scope of the criteria of interest. This study firstly explored the inter-factor correlations of the 10 personality aspects of the Work Personality Index (WPI) from an archival dataset of 4759 South African employees. An oblique lower-order model was specified to investigate the inter-factor correlations between personality aspects. Secondly, a dominance analysis was performed using a sequentially collected cross-sectional sample of 197 South African employees, to determine the relative weight that each of the 10 aspects carries when predicting five narrower dimensions of performance. The 10 aspects exhibited unique patterns in predicting narrower performance dimensions. Scientists and practitioners might, therefore, benefit from modelling relationships between personality aspects and narrower performance areas. In accordance, more nuanced selection decisions or development recommendations can be made that are specific to the work behaviours of interest.

**Contribution:**

The present study is the first to explore the criterion validity of the 10 personality aspects against five performance areas in the South African context. The study underscores the importance of a more nuanced understanding of the work-related implications of narrower personality characteristics, which have practical implications for both selection decisions and developmental recommendations within organisational settings.

## Introduction

A recent meta-analytical study by Sackett et al. (2021) suggests that personality, as a covert measure of integrity, is just as predictive of job performance as cognitive ability. Sackett et al. (2021) ranked personality in the fifth position in terms of criterion validity when compared to 24 other selection procedures. Meta-analytical studies conducted on the criterion validity of personality in South Africa similarly suggest that personality variables are important predictors of work performance (Van Aarde et al., 2017). However, Van Aarde et al. (2017) highlighted shortcomings in the way performance is conceptualised and measured in South Africa. For example, the analysis of the studies sampled for the meta-analysis by Van Aarde et al. (2017) demonstrated that performance evaluations (outcome variables) are mostly conducted for administrative purposes (promotion or remuneration), which negatively skews the performance scores. Counter to expectations, the Big Five trait *Conscientiousness* was found to display negligible validity in determining overall performance. Van Aarde et al. (2017) also discovered that the range of performance dimensions assessed in the studies was limited. Van Aarde et al. (2017) concluded that industrial psychology’s future credibility as a science in South Africa, especially studies of the validity of personality, will depend on the careful construction and judicious use of measures of individual work performance.

All the studies cited in the meta-analytical study conducted by Van Aarde et al. (2017) focussed on the Big Five factors of personality, namely *Openness to experience, Conscientiousness, Extraversion, Agreeableness, and Emotional stability*. A recent study conducted by Van Lill and Taylor (2021) replicated a hierarchical level of personality between the Big Five factors and personality facets according to the 10 personality aspects. DeYoung et al. (2007) and DeYoung (2015) were the first to provide theoretical arguments and empirical evidence for the existence of the 10 personality aspects, while Stanek and Ones (2018) highlighted its relevance for the workplace. A meta-analysis by Judge et al. (2013) revealed that the 10 personality aspects explained 10% more variance in overall job performance when compared to the Big Five factors. Judge et al. (2013) argue that the 10 personality aspects might more coherently represent the inter-factor correlations at the personality facet level, and better capture the nuanced relationships between personality and performance. Personality aspects might better represent the phenotypical patterns of thought, emotion, and behaviour than personality facets. The phenotypical patterns sprout from evidence from neurobiology and therefore reflect adaptive personality substructures that evolved in human beings because of environmental challenges (DeYoung, 2015; DeYoung et al., 2021).

Personality is predictive of performance and has a lower rate of adverse impact against previously disadvantaged groups in selection when compared to variables with similar criterion validity, such as cognitive ability (Outtz, 2002; Sackett et al., 2021; Van Lill & Coetzee, 2021). Consequently, personality assessments could be critical in helping South African employers make accurate and morally fair selection decisions. However, as alluded to by Van Aarde et al. (2017) and Van Lill and Taylor (2021), more nuanced measurement at the predictor level (personality) and the criterion level (work performance) could help professionals apply personality assessment results in a more judicious manner for selection and development purposes in the workplace. Given the current shortcomings regarding research on criterion validity with regard to personality in South Africa, the objective of the current study was to explore the relative weights that each of the 10 personality aspects explains with regard to five broad performance dimensions, namely *In-role-, Extra-role-, Adaptive-, Leadership-*, and *Counterproductive performance*, in the South African context.

## Manifestation of the 10 personality aspects among the facets of the work personality index

The manifestation of the 10 personality aspects based on the Work Personality Index (WPI) (Macnab & Bakker, 2014) had to be established. The WPI is a work-related personality measure that has been validated for the South African context (Macnab & Bakker, 2014). However, no prior studies have inspected the prevalence of the 10 personality aspects among the facets of the WPI.

**FIGURE 1:**
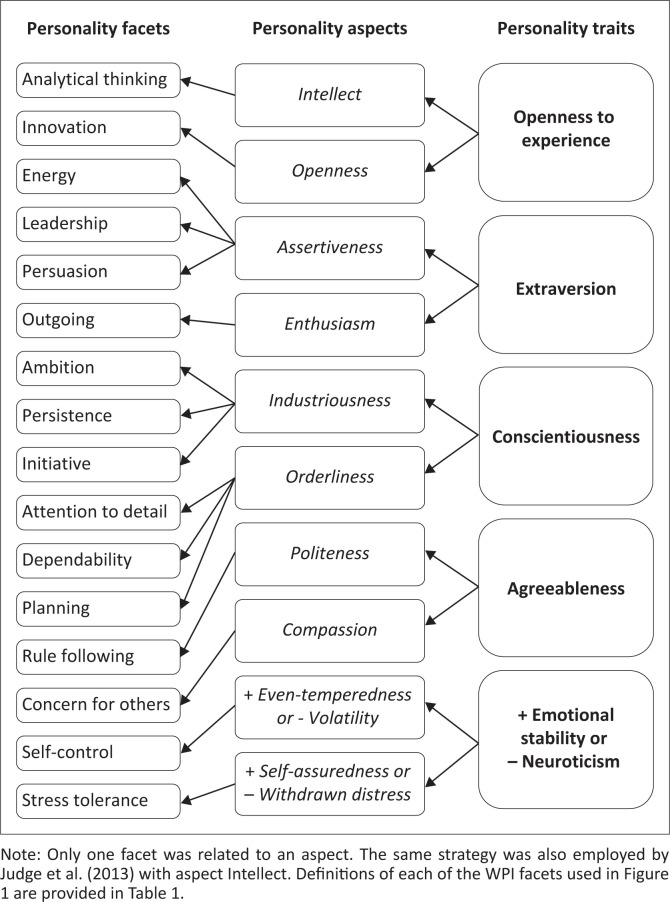
A non-statistical representation of the hierarchical structure of the work personality index (Macnab & Bakker, 2014), based on the Theoretical Arguments of DeYoung (2015) and Empirical Evidence of DeYoung et al. (2007) and Judge et al. (2013).

DeYoung (2015) argues that the 10 personality aspects better account for a personality substructure below the Big Five factors when compared to personality facets, where less consensus exists on the number and types of facets that make up personality. Personality aspects appear to have a stronger neurobiological foundation compared to personality facets and, as evolved human characteristics, are more self-evident adaptations to environmental challenges (DeYoung, 2015; DeYoung et al., 2021). The construction of the 10 aspects, as manifested in the facets of the WPI (Macnab & Bakker, 2014), were based on the Cybernetic Big Five Theory (CB5T) of DeYoung (2015) and empirical evidence of other personality measures (DeYoung et al., 2007; Judge et al., 2013; Van Lill & Taylor, 2021). The 10 aspects of personality, as a taxonomy of personality, can be seen as a ‘periodic table’ of individual differences, which could help build consensus regarding ‘first principles’ in the measurement of personality and its utility as a predictor of work-related outcomes (Woods & Anderson, 2016). An overview of the breakdown of the 10 personality aspects in relation to the WPI facets is provided in Figure 1.

DeYoung (2015) proposed the CB5T as an integrative framework to explain the reasons behind the atheoretical and empirically derived Big Five traits and 10 personality aspects. Cybernetic Big Five Theory holds that humans are goal-directed, self-regulating systems who aim to achieve desired future states. It is also argued that humans seek and integrate new information to revise their goals and strategies, enabling them to adapt to environmental changes. The traits *Openness* and *Extraversion* reflect the cognitive and behavioural exploration of new information and desirable alternatives, used to adapt existing goals and develop new strategies, and are sometimes collectively referred to as ‘*Plasticity*’ because of the traits’ shared covariance. The traits *Conscientiousness, Agreeableness,* and *Emotional stability*, jointly referred to as ‘*Stability*’ or ‘*Integrity*’, because of their shared covariance, reflect the self-regulation required to achieve current goals and execute the related strategies (DeYoung, 2015; Digman, 1997).

Trait *Openness to experience*, from a CB5T standpoint, is related to individual tendencies to cognitively explore and engage with new information in the world, prompting human beings to set alternative goals and devise creative and sufficiently complex strategies to achieve new goals (DeYoung, 2015). DeYoung et al. (2007) distinguish between two personality aspects related to trait *Openness to experience*, namely *Openness* and *Intellect. Openness* represents a tendency to make creative connections between new and chaotic information, which helps people gain novel insights. *Intellect*, by contrast, reflects a more abstract and analytical approach that enables individuals to evaluate the rationality of connections made between different pieces of information (DeYoung, 2015). In the current study, personality aspects *Intellect* and *Openness* were limited to the facets Analytical thinking and Innovation of the WPI (Macnab & Bakker, 2014), respectively.

**TABLE 1:** Definitions of different facets of the work personality index.

Facet	Definition
Ambition	Inclination to establish high standards, set challenging goals, and exert effort to attain success.
Analytical thinking	Inclination to meticulously analyse information and employ logical reasoning in addressing issues and problems.
Attention to detail	A proclivity to concentrate on details, strive for perfection, and approach tasks in a tidy and organised manner.
Concern for others	Level of sensitivity and understanding an individual possesses towards the needs and feelings of others.
Dependability	The degree to which an individual exhibits reliability and responsibility in fulfilling obligations.
Energy	Inclination to sustain a heightened level of energy and endure with vigour while working.
Initiative	Readiness to undertake new or additional work responsibilities and challenges.
Innovation	Level of creativity and open-mindedness exhibited when addressing work-related issues.
Leadership	Willingness to lead by taking charge of situations and providing opinions and directions to others.
Outgoing	Inclination for engaging with others and forming personal connections with people.
Persistence	Exemplifying the quality of persevering and surmounting obstacles in the completion of assigned duties.
Persuasion	Ease in negotiating, selling, influencing, and attempting to persuade others or alter their point of view.
Planning	Inclination to engage in work planning and adhere to the devised plan.
Rule following	Tendency to conform to rules and rigorously adhere to work regulations.
Self-control	The degree to which individuals maintain composure, regulate emotions, and manage anger.
Stress tolerance	Inclination to accept criticism and handle high-stress situations calmly and effectively.

Based on CB5T, the trait *Extraversion* reflects individual differences in reward sensitivity, which motivates human beings to behaviourally explore and engage with new goals (DeYoung, 2015). *Assertiveness* and *Enthusiasm* are distinguished as two aspects of *Extraversion* (DeYoung et al., 2007). Whereas *Assertiveness* drives individuals to behaviourally explore things external to themselves, *Enthusiasm* helps to reinforce their motivation to seek things outside of themselves through the enjoyment of newfound end states (DeYoung, 2015). *Assertiveness* aspect was inferred from the facets Energy, Leadership, and Persuasion, whereas *Enthusiasm* was represented by facet *Outgoing* of the WPI (Macnab & Bakker, 2014).

Based on CB5T, *Conscientiousness* reflects a tendency to self-regulate current behaviour, which helps individuals prioritise long-term survival goals (DeYoung, 2015). The two aspects related to *Conscientiousness* are *Industriousness* and *Orderliness* (DeYoung et al., 2007). *Industriousness* reflects the self-discipline to set aside short-term needs and put in the required effort to achieve long-term goals. *Orderliness*, in contrast, concerns self-set rules or adherence to rules set by others as boundaries that help individuals maintain their focus on socially appropriate and relevant goals (DeYoung, 2015). *Industriousness* aspect was based on the facets Ambition, Persistence, and Initiative, while *Orderliness* was derived from the facets Attention to detail, Dependability, and Planning of the WPI (Macnab & Bakker, 2014).

Humans are social, and the achievement of individual goals often needs to be balanced with collective needs. In the interpretive realm of CB5T, tendencies towards empathy and altruism, per the trait *Agreeableness*, trigger social bonding and reciprocity among human beings (DeYoung, 2015). DeYoung et al. (2007) highlight two personality aspects related to *Agreeableness*, namely *Politeness* and *Compassion. Politeness* aids individuals in cooperating within social groups through self-restraint of norm-violating impulses. *Compassion*, as a parallel strategy to operate in social groups, reinforces bonding through empathy for others (DeYoung, 2015). *Compassion* and *Politeness* were respectively represented by facets Concern for others and Rule following of the WPI (Macnab & Bakker, 2014).

*Neuroticism* (inversely referred to as *Emotional Stability* in the present study), according to CB5T (DeYoung, 2015), is a tendency to experience negative emotionality, which serves as a defence system to avoid undesirable end states, especially in the presence of a threat or uncertainty (DeYoung, 2015). Trait *Neuroticism*, according to DeYoung et al. (2007), can be divided into the aspects *Volatility* and *Withdrawn distress* – represented by the positively phrased *Even-temperedness* and *Self-assuredness. Volatility* represents an active defence in response to external threats by, for example, displaying aggression. *Withdrawn distress* also reflects a response to undesirable experiences but is more related to passive avoidance in response to uncertainty by, for example, experiencing anxiety. The behavioural inhibition system in the brain, with specific reference to the hippocampus, appears to be related to *Withdrawal* (DeYoung, 2015). Aspects *Even-temperedness* and *Self-assuredness* were theorised to, respectively, be related to the WPI facets Self-control and Stress tolerance (Macnab & Bakker, 2014).

## Criterion validity of the 10 personality aspects

While some researchers argue that personality traits are better predictors of performance than narrower facets (Ones & Viswesvaran, 1996), other scholars hold that narrower measures of personality are better predictors of narrow performance dimensions (Tett et al., 2003). Hough et al. (2015) recommend that the level of personality measured depends on the narrowness of the criteria of interest. Judge et al. (2013) suggest that the 10 personality aspects explain additional variance beyond the Big Five when predicting narrower dimensions of performance, such as task- and contextual performance. The purpose of the current study was to expand existing knowledge of how the 10 aspects, which are argued to more closely represent the phenotypical pattern of personality (DeYoung, 2015), are related to five narrower dimensions of individual work performance. Therefore, the present researchers looked at individual work performance across five dimensions, as proposed by Van Lill and Taylor (2022) in the Individual Work Performance Review, namely *In-role performance, Extra-role performance, Adaptive performance, Leadership performance*, and *Counterproductive performance*.

*In-role performance*, also referred to as ‘Task performance’, reflects the core tasks that employees must complete in their job (Van Lill & Taylor, 2022). Trait *Conscientiousness* is a consistent predictor of Task performance, independent of occupational characteristics, and is related to the self-restraint required to pursue non-immediate goals (Wilmot & Ones, 2021). Dudley et al. (2006) provided meta-analytical evidence, based on instruments such as the NEO Personality Inventory-Revised, that the facet Achievement striving (mean *r* = 0.13), which forms part of aspect *Industriousness*, might be the driving force in the correlation between trait *Conscientiousness* and Task performance. The meta-analysis of Judge et al. (2013), by contrast, demonstrated that the aspects *Industriousness* (*p* = 0.23) and *Orderliness* (*p* = 0.19) had the highest correlations with Task performance. *Assertiveness* (*p* = 0.15), perhaps because of its association with a behavioural drive to engage in things external to oneself, also appears to have a stronger relationship with *In-role performance*. Based on the findings of Judge et al. (2013), it was hypothesised that:

**H1:** The personality aspects *Industriousness, Orderliness*, and *Assertiveness* are the most dominant positive predictors of *In-role performance*.

*Extra-role performance*, akin to Contextual performance and Organisational citizenship behaviours, refers to actions that are not part of employees’ jobs, but are performed for the benefit of co-workers or team members (Van Lill & Taylor, 2022). The comparative strength of the predictive validity of personality aspects for *Extra-role performance* is less differentiated. The results of Judge et al. (2013) suggest a multitude of correlations between Contextual performance and the aspects *Industriousness* (*p* = 0.28), *Orderliness* (*p* = 0.27), *Compassion* (*p* = 0.14), *Politeness* (*p* = 0.16), *Volatility* (*p* = –0.21), *Assertiveness* (*p* = 0.15), and *Enthusiasm* (*p* = 0.15). In terms of relative strength, the aspects related to the trait *Conscientiousness* appear to have more dominant relationships with *Contextual performance* (Wilmot, 2017). Pletzer et al. (2021), in a meta-analytical study based on HEXACO Inventory, found that the facet Diligence (associated with the aspect *Industriousness*) appears to have a much stronger relationship with Organisational citizenship behaviour, compared to facets associated with the aspect *Orderliness*, namely Organisation, Perfection, and Prudence. Higher *Industriousness* might help individuals to maintain the direction of their focus on longer-term goals, while also increasing the intensity and persistence of their behaviour – reflected in actions beyond what is normally required – to achieve their aspirations (DeYoung, 2015; Judge & Ilies, 2002).

*Extra-role performance*, as measured by the Individual Work Performance Review (IWPR) (Van Lill & Taylor, 2022), also includes dimensions that go beyond typical conceptualisations of contextual performance, namely Self-development and Innovation (George & Brief, 1992; Podsakoff et al., 2000). Initiating learning and experimenting with new ways of doing things might require a greater tendency to cognitively explore and analyse new information. Consequently, it was hypothesised that:

**H2:** The personality aspects *Industriousness, Intellect*, and *Openness* are the most dominant positive predictors of *Extra-role performance*.

*Adaptive performance* reflects an employee’s ability to adapt to crises or deal with novelty and ambiguity (Van Lill & Taylor, 2022). A meta-analysis conducted by Huang et al. (2014) suggested that traits Ambition (p = 0.16) and Adjustment (*p* = 0.14), related to the traits *Extraversion* and *Neuroticism*, respectively (Hogan & Hogan, 2007), have the strongest relationships with *Adaptive performance*. Employees who score higher on the trait Ambition, with the aspect *Assertiveness* being the closest representation of the trait (Hogan & Hogan, 2007), might proactively modify their objectives in response to change, to attain greater social status. Higher Adjustment, related to aspect *Self-assuredness* (Hogan & Hogan, 2007), might assist employees in functionally reacting (a passive response) to the environmental threats posed by change and uncertainty (Huang et al., 2014). Based on the meta-analytical evidence, it was hypothesised that:

**H3:** The personality aspects *Assertiveness* and *Self-assuredness* are the most dominant positive predictors of *Adaptive performance*.

*Leadership performance* refers to the ability to effectively influence co-workers towards the achievement of common goals (Van Lill & Taylor, 2022). The most dominant correlates of Leadership effectiveness appear to be traits *Extraversion* (Judge et al., 2002; p = 0.24), *Openness* (Judge et al., 2002; *p* = 0.24), and *Conscientiousness* (Judge et al., 2002; *p* = 0.16). The findings are also corroborated by the meta-analytical evidence of Wilmot (2017) and Wilmot and Ones (2021). DeYoung et al. (2007), based on the work of Saucier et al. (2005), argue that the covariation between the aspects *Assertiveness, Intellect*, and *Industriousness* might represent a composite trait, referred to as ‘*Heroism*’. *Heroism* is defined as the exceptionality or competence of an employee, which provides signals of the individual’s worthiness of being followed. Based on the concept of heroism and current meta-analytical findings, it was hypothesised that:

**H4:** The personality aspects *Assertiveness, Intellect*, and *Industriousness* are the most dominant positive predictors of *Leadership performance*.

*Counterproductive performance* encompasses those actions that can negatively impact others or prevent teams or organisations from achieving common goals (Van Lill & Taylor, 2022). Personality-based *Integrity*, also referred to as meta-trait ‘*Stability*’, appears to be the most dominant meta-analytical predictor of *Counterproductive performance* (Ones et al., 2007; Pletzer et al., 2020; Wilmot, 2017). *Conscientiousness* (Wilmot, 2017; *p* = –0.39), *Agreeableness* (Wilmot, 2017; *p* = –0.45), and *Emotional stability* (Wilmot, 2017; *p* = –0.30) make up personality-based integrity and are the primary traits that predict *Counterproductive performance*. Facet-level predictors of *Counterproductive performance*, as identified by Pletzer et al. (2020) and Morris et al. (2015), reveal a distinctive pattern of relationships with *Counterproductive performance*. Individuals’ self-regulating or impulse control tendencies, aimed at preventing norm-violating behaviours, might best explain future *Counterproductive performance*. Regarding more self-regulative (or rule-following) aspects in the CB5T (DeYoung, 2015), it was hypothesised that:

**H5:** The personality aspects *Orderliness, Politeness*, and *Even-temperedness* are the most dominant negative predictors of *Counterproductive performance*.

## Research design

### Research approach

A large archival dataset was first leveraged to explore the structural validity of the 10 aspects based on the WPI, in South Africa. A cross-sectional, quantitative research design was sequentially utilised to investigate the concurrent validity of the WPI against the performance criteria of interest. A cross-sectional design enabled a nuanced view of the nature of personality at a single point in time, and an efficient quantitative exploration of relationships between a large set of personality and performance variables across different organisational contexts (Spector, 2019). Multiple sources, namely self-ratings on personality and managerial ratings of performance, further aided the rigour of the cross-sectional design by accounting for a source of method variance (Podsakoff et al., 2012), namely the leniency bias associated with self-ratings of performance (Aguinis, 2019; Spector, 2019; Van Lill & Van der Merwe, 2022).

### Research method

#### Research participants

Archival data from 4759 South African employees were collected between 2018 and 2021, which were used to inspect the structure of the relationships between and discriminant validity of the 10 personality aspects based on the WPI (Macnab & Bakker, 2014). The mean age of employees was 34.67 years (standard deviation [s.d.] = 8.89 years). Most of the employees who disclosed their ethnicity self-identified as black African (*n* = 1898; 49%), followed by white (*n* = 1287; 33%), coloured (*n* = 448; 11%), and Asian and/or Indian (*n* = 268; 7%). The sample comprised more men (*n* = 2586; 54%) than women (*n* = 2173; 46%). The researchers computed the power for the test model (degrees of freedom [*df*] = 8201), based on the computer software developed by Preacher and Coffman (2006). The models returned a value of unity, which suggested that an incorrect model would be correctly rejected (α = 0.05; null root mean square error of approximation [RMSEA] = 0.05; alternative RMSEA = 0.08).

A total of 197 performance ratings of South African employees, who were also administered with the WPI (Macnab & Bakker, 2014), were completed by managers in two participating organisations, via a census or a stratified sampling strategy. Concurrent performance data were collected as a separate process from the archival dataset, sampled at a later stage of data collection, and were smaller than the overarching archival dataset. However, the data from the WPI were still included in the overall archival dataset to explore the structural validity of the 10 aspects. The sample represented the financial and professional services sectors. The mean age of employees was 36.68 years (s.d. = 6.82 years). Most of the employees who disclosed their ethnicity self-identified as white (*n* = 95; 49%), followed by black African (*n* = 46; 24%), Indian (39; 20%), coloured (*n* = 13; 7%), and Asian (*n* = 2; 1%). The sample comprised more women (*n* = 118; 61%) than men (*n* = 77; 39%). Most of the employees were registered professionals (*n* = 77; 39%), followed by skilled employees (*n* = 60; 31%), low-level managers (*n* = 39; 20%), mid-level managers (*n* = 15; 8%), and top-level managers (*n* = 4; 2%). The statistical power required for multiple regression with 10 predictors was calculated using G*Power (Faul et al., 2007), which suggested that 172 participants should be sufficient (α = 0.05; Power = 0.80) to detect an effect size of 0.10.

#### Measuring instruments

Data were collected using two instruments, the Second Edition of the WPI (Macnab & Bakker, 2014) and the IWPR (Van Lill & Taylor, 2022). The WPI (Macnab & Bakker, 2014) consists of 22 scales, of which 21 provide information about an individual’s personality make-up. The 22nd scale indicates motivational distortion. In a 2022 South African research supplement for the WPI (Hayes & Van Lill, 2022), the reliability (*n* = 5078) for the 16 scales used in the present study ranged from ω = 0.76 to ω = 0.90. The IWPR (Van Lill & Taylor, 2022) measures five broad performance dimensions: *In-role-, Extra-role-, Adaptive-, Leadership-*, and *Counterproductive performance*. Each of these broad dimensions consists of four narrower dimensions of performance. For a further discussion of these dimensions, see Van Lill and Taylor (2022). The internal consistency for the narrow dimensions ranged from ω = 0.87 to ω = 0.97 for a sample of 448 South African participants across six organisations (Van Lill & Taylor, 2022).

#### Research procedure

Data were collected via online assessments using the WPI (Macnab & Bakker, 2014) in different workplace settings as part of several projects undertaken by the JVR Africa Group. Performance data were concurrently collected from appointed employees’ managers using the IWPR (Van Lill & Taylor, 2022).

#### Statistical analysis

The first important step in the analysis was to obtain a descriptive overview of the data by determining the inter-factor correlations between the 10 aspects, the mean item score, and s.d. for each personality aspect, and the internal consistency reliability of each aspect. The inter-factor correlations were calculated using oblique lower-order confirmatory factor analysis (CFA). Cronbach’s alpha (Cronbach, 1951) and McDonald’s omega (McDonald, 1999) were calculated to gain an impression of the internal consistency reliabilities of the 10 aspects. Cronbach’s alpha coefficient and McDonald’s omega coefficient were calculated using Version 0.5–6 of the semTools package in R (Jorgensen et al., 2022).

Diagonally weighted least squares (DWLS) estimation, with robust errors, was performed to inspect the fit of all the models specified in the current study (DiStefano & Morgan, 2014; Li, 2016). Diagonally weighted least squares with robust errors was deemed appropriate because of the larger sample (*n* > 500) used, non-normal distributions of the data, and the ordinal nature of the rating scales with five qualitative anchors (DiStefano & Morgan, 2014; Li, 2016). The multivariate skewness (2 762 044.90; *p* < 0.001) and kurtosis (519.37; *p* < 0.001) for the entire set of 131 items (excluding the Social desirability scale) suggested that the data were non-normally distributed. Model-data fit was considered acceptable if the RMSEA and standardised root mean square residual (SRMR) were ≤ 0.08 (Brown, 2015; Browne & Cudeck, 1992) and the comparative fit index (CFI) and Tucker-Lewis index (TLI) were > 0.95 (Brown, 2015; Hu & Bentler, 1999). Even when CFIs display a marginally good fit to the data (CFI and TLI in the range of 0.90–0.95), models might still be considered to display an acceptable fit if other indices, that is, SRMR and RMSEA are within the acceptable range (Brown, 2015).

The interpretation of the relative weight of multiple regression coefficients might be incorrect when multi-collinearity exists between the predictive variables (Nimon & Oswald, 2013). Personality variables are not theorised to be uncorrelated, but appear to share common variance, because of the existence of the superordinate meta-traits, namely *Stability* and *Plasticity* (DeYoung, 2015). Dominance analysis was performed with Version 2.0-3 of the yhat package in R (Nimon et al., 2021; Nimon & Oswald, 2013). This enabled the researchers to determine the relative weights that each of the personality variables would carry as predictors of the relevant performance dimensions identified.

### Ethical considerations

Ethical approval for the study was obtained from The Department of Industrial Psychology and People Management’s Research Ethics Committee members at the University of Johannesburg (reference number: IPPM-2022-599). The study was low in risk, but precautions were taken to ensure that participation was voluntary and anonymous, no harm was caused, the questions were answered truthfully, and informed consent was given to use the results for research purposes. Participants were informed about the nature of the measurement, voluntary participation, benefits of participation, anonymity of their personal data, and that the data would be used for research purposes.

## Results

### Descriptive statistics

Table 2 provides the mean item score and s.d. for each personality aspect of the WPI (Macnab & Bakker, 2014), along with the alpha and omega reliability estimates and standardised inter-factor correlations of the 10 aspects. The inter-factor correlations were obtained by conducting an oblique lower-order confirmatory factor model. Aspects *Assertiveness, Industriousness*, and *Orderliness* were specified as higher-order factors in the oblique lower-order model. The upper limit (UL) of the inter-factor correlations is provided above the diagonal. The fit statistics for the oblique lower-order confirmatory factor model of the entire WPI (χ^2^ [*df*] = 179 424.96 [8201]; CFI = 0.93; TLI = 0.92; SRMR = 0.07; RMSEA = 0.07 [0.07; 0.07]), based on the South African dataset, were satisfactory (Brown, 2015; Hu & Bentler, 1999).

**TABLE 2:** Inter-factor correlations, reliabilities, means and standard deviations of the 10 personality aspects based on the work personality index.

Dimensions	Mean	s.d.	Alpha	Omega	OPE	INT	ASS	ENT	IND	ORD	POL	COM	EVE	SEL
Openness (OPE)	4.15	0.49	0.77	0.80	-	0.68	0.62	0.33	0.73	0.68	0.30	0.45	0.39	0.49
Intellect (INT)	3.85	0.54	0.80	0.78	0.66*	-	0.79	0.43	0.72	0.53	0.18	0.39	0.37	0.51
Assertiveness (ASS)	3.66	0.44	0.88	0.75	0.59*	0.78*	-	0.74	0.91	0.63	0.40	0.40	0.42	0.72
Enthusiasm (ENT)	3.47	0.52	0.70	0.70	0.30*	0.40*	0.72*	-	0.57	0.43	0.38	0.45	0.51	0.57
Industriousness (IND)	4.03	0.36	0.82	0.73	0.71*	0.70*	0.89*	0.54*	-	0.87	0.52	0.52	0.57	0.76
Orderliness (ORD)	4.05	0.42	0.87	0.80	0.66*	0.50*	0.61*	0.40*	0.86*	-	0.73	0.45	0.60	0.60
Politeness (POL)	3.88	0.65	0.86	0.86	0.27*	0.15*	0.37*	0.36*	0.50*	0.71*	-	0.28	0.50	0.43
Compassion (COM)	4.04	0.51	0.78	0.80	0.42*	0.36*	0.37*	0.42*	0.49*	0.42*	0.25*	-	0.55	0.40
Even-tempered (EVE)	3.73	0.59	0.71	0.72	0.36*	0.34*	0.39*	0.48*	0.54*	0.58*	0.47*	0.52*	-	0.84
Self-assured (SEL)	3.87	0.57	0.80	0.81	0.46*	0.49*	0.70*	0.55*	0.74*	0.58*	0.40*	0.37*	0.82*	-

Note: Omega hierarchical coefficients are provided for *Assertiveness, Industriousness*, and *Orderliness*. The upper limit (UL) of the inter-factor correlations is provided above the diagonal.

* , *p* < 0.05.

At a first glance, the inter-factor correlations below the diagonal in Table 2 suggest that most of the personality aspects related to the same trait display a fair degree of conceptual overlap, except for *Compassion* and *Politeness*. Few facets mapped onto *Politeness*; consequently, an empirical compromise had to be made by assigning a facet, namely Rule following, in favour of the facet’s theoretical overlap with *Politeness*. The facets Compliance (Costa & McCrae, 1992; NEO Personality Inventory-Revised) and Morality (Hofstee et al., 1992; Abridged Big Five-Dimensional Circumplex) have been assigned to the trait *Agreeableness* in the past. It is also worth noting that the 10 personality aspects, in general, are correlated with each other. This corresponds with the argument that covariation, beyond the Big Five traits, can be explained by meta-traits (DeYoung, 2015; Digman, 1997).

The UL of 93% of the inter-factor correlations in Table 2 was below the cut-off of < 0.80 proposed by Rönkkö and Cho (2020) and, therefore, most of the narrow dimensions of personality displayed sufficient discriminant validity. Rönkkö and Cho (2020) consider inter-factor correlations of 0.80 ≤ UL < 0.90 as marginally problematic; 7% of the UL correlations in Table 2, based on this guideline, had lower discriminant validity. However, 4% of the UL correlations with marginal discriminant validity were between personality aspects that have a trait in common, namely Industriousness and Orderliness and Even-tempered and Self-assured. The remaining 3%, namely between *Industriousness* and *Assertiveness*, were not unexpected, and have been theorised and proven to be related to each other in prior studies (DeYoung et al., 2007).

### Dominance analysis

The notable degree of overlap between the traits served as confirmation of the necessity to conduct a dominance analysis to determine the relative weights of the 10 personality aspects’ relationship with the dimensions of performance. The coefficients of the analyses for each of the five performance dimensions are reported in Table 3 to Table 7.

**TABLE 3:** Relative importance of the work personality index’s five personality traits for in-role performance.

Metric	INT	OPE	ASS	ENT	IND	ORD	POL	COM	EVE	SEL
*B*	0.19	−0.35	−0.05	−0.02	0.34†	−0.02	−0.02	−0.06	0.23	−0.10
Beta	0.08	−0.17	−0.07	−0.01	0.38†	−0.02	−0.02	−0.03	0.10	−0.06
*R*	0.19†	0.03	0.10	0.04	0.28†	0.16	0.09	0.04	0.12	0.09
*r_s_*	0.57†	0.08	0.31	0.12	0.84†	0.48	0.29	0.11	0.37	0.26
$r_{s}^{2}$	0.32	0.01	0.10	0.01	0.71	0.23	0.08	0.01	0.14	0.07
Unique	< 0.01	0.02	< 0.01	< 0.01	0.05	< 0.01	< 0.01	< 0.01	0.01	< 0.01
Common	0.03	−0.02	0.01	< 0.01	0.03	0.03	0.01	< 0.01	0.01	0.01
GenDom	0.02	0.01	0.01	< 0.01	0.06	0.01	< 0.01	< 0.01	0.01	< 0.01
Rescaled (%)	14	9	5	1	56	6	2	1	5	2
Position	2	3	5	9	1	4	7	10	6	8
Pratt	0.02	−0.01	−0.01	< 0.01	0.10	< 0.01	< 0.01	< 0.01	0.01	−0.01
RLW	0.02	0.01	0.01	< 0.01	0.05	0.01	< 0.01	< 0.01	0.01	< 0.01

Unique, uniqueness coefficient; common, communality coefficient; GenDom, general dominance weight; Rescaled, rescaled general dominance weight; Pratt, Pratt measure; RLW, relative weights; INT, Intellect; OPE, Openness; ASS, Assertiveness; ENT, Enthusiasm; IND, Industriousness; ORD, Orderliness; POL, Politeness; COM, Compassion; EVE, Even-temperedness; SEL, Self-assuredness.

† , Confidence intervals do not include zero for *b*, Beta, *r*, and *r_s_*.

**TABLE 4:** Relative importance of the work personality index’s five personality traits for extra-role performance.

Metric	INT	OPE	ASS	ENT	IND	ORD	POL	COM	EVE	SEL
*B*	0.41	−0.21	−0.04	−0.07	0.42†	−0.23	−0.18	−0.02	0.34	−0.18
Beta	0.13	−0.08	−0.05	−0.03	0.35†	−0.24	−0.10	−0.01	0.12	−0.09
*R*	0.18†	0.09	0.06	−0.01	0.19†	−0.08	−0.13	−0.04	0.04	0.01
*r_s_*	0.53†	0.27	0.17	−0.02	0.55†	−0.24	−0.38	−0.11	0.11	0.02
$r_{s}^{2}$	0.28	0.07	0.03	< 0.01	0.31	0.06	0.14	0.01	0.01	< 0.01
Unique	0.01	< 0.01	< 0.01	< 0.01	0.05	0.02	< 0.01	< 0.01	0.01	< 0.01
Common	0.03	0.01	< 0.01	< 0.01	−0.01	−0.01	0.01	< 0.01	−0.01	< 0.01
GenDom	0.02	< 0.01	< 0.01	< 0.01	0.05	0.02	0.02	< 0.01	< 0.01	< 0.01
Rescaled (%)	18	3	3	1	38	17	13	1	3	3
Position	2	5	7	9	1	3	4	10	6	8
Pratt	0.02	−0.01	< 0.01	< 0.01	0.07	0.02	0.01	< 0.01	< 0.01	< 0.01
RLW	0.02	< 0.01	0.01	< 0.01	0.04	0.02	0.02	< 0.01	< 0.01	< 0.01

Unique, uniqueness coefficient; common, communality coefficient; GenDom, general dominance weight; Rescaled, rescaled general dominance weight; Pratt, Pratt measure; RLW, relative weights; INT, Intellect; OPE, Openness; ASS, Assertiveness; ENT, Enthusiasm; IND, Industriousness; ORD, Orderliness; POL, Politeness; COM, Compassion; EVE, Even-temperedness; SEL, Self-assuredness.

† , Confidence intervals do not include zero for *b*, Beta, *r*, and *r_s_*.

**TABLE 5:** Relative importance of the work personality index’s five personality traits for adaptive performance.

Metric	INT	OPE	ASS	ENT	IND	ORD	POL	COM	EVE	SEL
*B*	0.16	−0.14	0.02	−0.10	0.30†	−0.16	−0.21	0.20	0.20	0.10
Beta	0.06	−0.06	0.03	−0.05	0.28†	−0.18	−0.13	0.09	0.07	0.06
*R*	0.18†	0.12	0.16	0.07	0.22†	−0.02	−0.07	0.06	0.10	0.13
*r_s_*	0.56†	0.37	0.51	0.23	0.69†	−0.06	−0.21	0.18	0.33	0.42
$r_{s}^{2}$	0.31	0.14	0.26	0.05	0.48	< 0.01	0.04	0.03	0.11	0.17
Unique	< 0.01	< 0.01	< 0.01	< 0.01	0.03	0.01	0.01	0.01	< 0.01	< 0.01
Common	0.03	0.01	0.03	< 0.01	0.02	−0.01	< 0.01	< 0.01	0.01	0.02
GenDom	0.01	< 0.01	0.01	0.00	0.04	0.01	0.01	< 0.01	0.01	0.01
Rescaled (%)	12	3	8	2	36	12	12	3	5	6
Position	2	8	5	10	1	3	4	9	7	6
Pratt	0.01	−0.01	0.01	< 0.01	0.06	< 0.01	0.01	0.01	0.01	0.01
RLW	0.01	< 0.01	0.01	< 0.01	0.03	0.01	0.01	< 0.01	0.01	0.01

Unique, uniqueness coefficient; common, communality coefficient; GenDom, general dominance weight; Rescaled, rescaled general dominance weight; Pratt, Pratt measure; RLW, relative weights; INT, Intellect; OPE, Openness; ASS, Assertiveness; ENT, Enthusiasm; IND, Industriousness; ORD, Orderliness; POL, Politeness; COM, Compassion; EVE, Even-temperedness; SEL, Self-assuredness.

† , Confidence intervals do not include zero for *b*, Beta, *r*, and *r_s_*.

**TABLE 6:** Relative importance of the work personality index’s five personality traits for leadership performance.

Metric	INT	OPE	ASS	ENT	IND	ORD	POL	COM	EVE	SEL
*B*	0.28	−0.04	0.02	0.02	0.45†	−0.23	−0.37	0.18	0.21	−0.16
Beta	0.08	−0.01	0.02	0.01	0.35†	−0.22	−0.19	0.06	0.06	−0.07
*R*	0.19†	0.16†	0.14	0.07	0.22†	−0.10	−0.17†	0.02	0.02	0.02
*r_s_*	0.50†	0.40†	0.37	0.17	0.58†	−0.26	−0.43†	0.05	0.04	0.06
$r_{s}^{2}$	0.25	0.16	0.13	0.03	0.34	0.07	0.19	< 0.01	< 0.01	< 0.01
Unique	< 0.01	< 0.01	< 0.01	< 0.01	0.05	0.02	0.02	< 0.01	< 0.01	< 0.01
Common	0.03	0.02	0.02	< 0.01	0.01	−0.01	0.01	< 0.01	< 0.01	< 0.01
GenDom	0.02	0.01	0.01	< 0.01	0.05	0.02	0.03	< 0.01	< 0.01	< 0.01
Rescaled (%)	12	5	6	1	35	17	21	1	1	1
Position	4	6	5	7	1	3	2	8	10	9
Pratt	0.02	< 0.01	< 0.01	< 0.01	0.08	0.02	0.03	< 0.01	< 0.01	< 0.01
RLW	0.02	0.01	0.01	< 0.01	0.05	0.02	0.03	< 0.01	< 0.01	< 0.01

Unique, uniqueness coefficient; common, communality coefficient; GenDom, general dominance weight; Rescaled, rescaled general dominance weight; Pratt, Pratt measure; RLW, relative weights; INT, Intellect; OPE, Openness; ASS, Assertiveness; ENT, Enthusiasm; IND, Industriousness; ORD, Orderliness; POL, Politeness; COM, Compassion; EVE, Even-temperedness; SEL, Self-assuredness.

† , Confidence intervals do not include zero for *b*, Beta, *r*, and *r_s_*.

**TABLE 7:** Relative importance of the work personality index’s five personality traits for counterproductive performance.

Metric	INT	OPE	ASS	ENT	IND	ORD	POL	COM	EVE	SEL
*B*	0.09	0.12	−0.06	0.18	−0.20†	0.03	0.05	−0.07	−0.18	0.07
Beta	0.05	0.08	−0.12	0.15	−0.29†	0.06	0.05	−0.05	−0.11	0.06
*R*	−0.10	−0.05	−0.14	< 0.01	−0.22†	−0.08	−0.03	−0.05	−0.09	−0.06
*r_s_*	−0.36	−0.18	−0.53	< 0.01	−0.81†	−0.30	−0.13	−0.17	−0.33	−0.23
$r_{s}^{2}$	0.13	0.03	0.29	< 0.01	0.65	0.09	0.02	0.03	0.11	0.05
Unique	< 0.01	< 0.01	0.01	0.01	0.03	< 0.01	< 0.01	< 0.01	0.01	< 0.01
Common	0.01	< 0.01	0.02	−0.01	0.02	0.01	< 0.01	< 0.01	< 0.01	< 0.01
GenDom	< 0.01	< 0.01	0.01	0.01	0.04	< 0.01	< 0.01	< 0.01	< 0.01	< 0.01
Rescaled (%)	4	3	17	10	53	3	1	1	6	3
Position	5	6	2	3	1	7	9	10	4	8
Pratt	−0.01	< 0.01	0.02	< 0.01	0.06	−0.01	< 0.01	< 0.01	0.01	< 0.01
RLW	< 0.01	< 0.01	0.01	0.01	0.03	< 0.01	< 0.01	< 0.01	0.01	< 0.01

Unique, uniqueness coefficient; common, communality coefficient; GenDom, general dominance weight; Rescaled, rescaled general dominance weight; Pratt, Pratt measure; RLW, relative weights; INT, Intellect; OPE, Openness; ASS, Assertiveness; ENT, Enthusiasm; IND, Industriousness; ORD, Orderliness; POL, Politeness; COM, Compassion; EVE, Even-temperedness; SEL, Self-assuredness.

† , Confidence intervals do not include zero for *b*, Beta, *r*, and *r_s_*.

Only the confidence intervals of coefficients for *Industriousness*, in all the tables reporting regression coefficients, did not include zero, and were therefore statistically significant. However, as stated by Cohen (1990, p. 1310), ‘The primary product of a research inquiry is one or more measures of effect size, not *p* values’. Consideration is, therefore, primarily given to the effect sizes reported in Table 3 to Table 7, which is more in accord with best practice in reporting findings to avoid publication bias associated with only reporting significant findings (Funder & Ozer, 2019). According to Funder and Ozer (2019), an effect-size *r* of 0.05 suggests a minimal impact on the explanation of individual events, yet it could have significant consequences in the long term. When the effect size is 0.10, it remains small on the level of single events but holds the potential for greater ultimate significance. With an effect-size *r* of 0.20, the impact is of medium size, providing some explanatory and practical utility even in the short run, making it more noteworthy. A larger effect-size *r* of 0.30 indicates a substantial and potentially powerful effect both in the short and long term. It is proposed that a very large effect size (*r* = 0.40 or greater) in psychological research is likely an exaggeration and seldom observed in large samples or replications.

Confirming Hypothesis 1, *Industriousness* (*r* = 0.28; rescaled = 56%) had the most dominant effect on *In-role performance*, as reported in Table 2. *Orderliness* (*r* = 0.16; rescaled = 6%) and *Assertiveness* (*r* = 0.10; rescaled = 5%), contrary to expectations, were the fourth- and fifth-most dominant predictors of *In-role performance*, but more substantive than *Openness* when considering the size of the correlations. *Intellect* (*r* = 0.19; rescaled = 14%), unexpectedly, was the second-most dominant predictor of *In-role performance*.

*Industriousness* (*r* = 0.19; rescaled = 38%) had the most dominant relationship with *Extra-role performance*, per the coefficients in Table 4, which provided support for Hypothesis 2. Contrary to expectations, aspects related to *Openness to experience*, namely *Intellect* (*r* = 0.18; rescaled = 18%) and *Openness* (*r* = 0.09; rescaled = 3%), were in the fourth and fifth positions as predictors of *Extra-role performance*. However, when only considering positive relationships, the hypothesised pattern of dominant predictors, with *Intellect, Openness*, and *Industriousness* in the second, third, and first positions, could be confirmed.

*Industriousness* (*r* = 0.22; rescaled = 36%) and *Intellect* (*r* = 0.18; rescaled = 12%), contrary to Hypothesis 3, were the strongest positive predictors of *Adaptive performance*, as evidenced by the coefficients reported in Table 5. Both *Assertiveness* (*r* = 0.16; rescaled = 8%) and *Self-assuredness* (*r* = 0.13; rescaled = 6%) were relevant positive predictors, in the fifth and sixth position, but not as dominant as the other two aspects. However, when considering only positive relationships, *Assertiveness* and *Self-assuredness* would take third and fourth positions, respectively, as dominant predictors of *Adaptive performance*.

Confirming Hypothesis 4, *Industriousness* (*r* = 0.22; rescaled = 35%) was the most dominant predictor of *Leadership performance* (see Table 6). *Intellect* (*r* = 0.19; rescaled = 12%) and *Assertiveness* (*r* = 0.14; rescaled = 6%), by contrast, only attained the fourth and fifth positions in terms of predictive validity. However, when only considering the positive relationships, the hypothesised pattern of dominant predictors was confirmed, with *Assertiveness, Intellect*, and *Industriousness* in the third, second, and first positions in terms of their predictive validity for *Leadership performance*.

*Industriousness* (*r* = -0.22; rescaled = 53%), *Assertiveness* (*r* = -0.14; rescaled = 17), and *Intellect* (*r* = -0.10; rescaled = 4%) were the most dominant negative predictors of *Counterproductive performance* (see Table 7). *Orderliness* (*r* = -0.08; rescaled = 3%), *Politeness* (*r* = -0.03; rescaled = 1%), and *Even-temperedness* (*r* = -0.09; rescaled = 6%), contrary to expectations, were only the seventh-, ninth-, and fourth-most dominant predictors of *Counterproductive performance*, but in the hypothesised direction.

## Discussion

*Industriousness* and *Intellect* emerged as the most dominant predictors of positive dimensions related to work performance, independent of the broad performance dimensions studied. The sample of the present study consisted of mainly professionals (*n* = 77; 39%). However, when considering a broader definition of the term ‘professional’ (not just individuals registered with a professional board, but also those with a high level of education), used in prior meta-analytical studies (Wilmot & Ones, 2021), many of the respondents in the present study classified as skilled employees (*n* = 60; 31%), and could also be viewed as professionals. Wilmot and Ones (2021) provided meta-analytical evidence to suggest that *Openness to experience* (*p* = 0.20; rescaled = 66%) and *Conscientiousness* (*p* = 0.14; rescaled = 29%) are the strongest predictors of performance in professional jobs. The present study, while not intended, highlights that *Intellect* and *Industriousness* might be the primary drivers behind the criterion validity of *Openness to experience* and *Conscientiousness* among a larger cohort of professionals.^1^

The evidence points to the importance of uncoupling aspects in traits such as *Extraversion, Openness to experience, Conscientiousness, and Emotional stability. Conscientiousness* tends to generalise across occupations as a consistent trait-based predictor of work performance (Wilmot & Ones, 2021). In accordance with Van Aarde et al.’s (2017) expectations, an aspect related to *Conscientiousness* is a consistent predictor of different performance outcomes. However, the present findings suggest that *Orderliness*, when uncoupled from *Industriousness*, might, in many cases, have a negative relationship with performance outcomes, except for *In-role performance*.

*Assertiveness*, when uncoupled from *Enthusiasm*, shared greater overlap with *Intellect* and *Industriousness*, which replicated the pattern of correlations observed by DeYoung et al. (2007). DeYoung et al. (2007) suggested that the covariation could be explained by a composite trait that captures something related to admirable characteristics in individuals. In the context of the present work-related sample, the proclivity to critically analyse information (*Intellect*), prioritise long-term goals (*Industriousness*), and behavioural motivation to pursue new goals (*Assertiveness*) appear to be markers of high potential. This combination of markers of high potential, as inferred from the aspect level, appears to be particularly salient when predicting *Leadership performance*.

*Even-temperedness* and *Self-assuredness* further highlight the unique relationships that personality aspects have with work performance, compared to the constituent trait *Emotional stability*. Whereas *Self-assuredness* appears to be particularly important for *Adaptive performance, Even-temperedness* appears to have stronger relations with *In-role-, Extra-role-*, and *Counterproductive performance. Even-temperedness*, in line with the findings of Judge et al. (2013), appears to be a more generalisable predictor across dimensions of work performance, perhaps because of the proclivity to self-regulate immediate emotional impulses in the pursuit of larger work-related goals.

The present study suggests a fair degree of variability in the degree to which personality aspects predict narrower performance outcomes. In heeding Van Aarde et al.’s (2017) recommendation, it might be sensible for practitioners to be more prudent in their choice of personality dimensions chosen when making decisions about people based on narrower performance criteria. Uncoupling aspects related to broad traits reveal unique relationships with performance that are not always visible at the trait level. Aspects, as more coherent representations of facets (DeYoung, 2015), might be a level of personality measurement that is broad enough to make reliable inferences about future behaviour for selection decisions, based on targeted performance dimensions, and narrow enough to make tailored personality-based development suggestions (Judge et al., 2013).

### Limitations and recommendations for future research

Even though meaningful relationships between personality and performance, by most hypotheses, were found in the present study, a few limitations should be mentioned. The present study mainly relied on a relatively small sample representing professional employees, which might have skewed the relevance of the personality dimensions investigated. Larger samples might enable the exploration of more precise and reliable effects (Funder & Ozer, 2019). Future studies could also consider the relative predictive validity of personality aspects for other job families, such as technical (requiring hands-on training), clerical (administrative), marketing (sales), military, healthcare, customer service, law enforcement, and management positions. Occupational category might play an important role in dominance of the role that personality aspects play when predicting different performance outcomes (Wilmot & Ones, 2021). For example, being more Orderly might play a more dominant role in predicting In-role performance with clerical positions. Furthermore, some aspects were based on only one facet, which is not uncommon (Judge et al., 2013), but future studies could consider measures of the 10 aspects that provide a richer representation at the facet level. Aspect *Politeness*, for example, might be a stronger negative predictor of *Counterproductive performance* in studies that measure more encompassing representations of the aspect.

It was assumed that the relationship between personality and some of the performance outcomes was linear. However, it is entirely possible that more complex relationships could exist between personality variables and specific performance outcomes. For example, Van Zyl and De Bruin (2018) have illustrated how linear regression methods can overestimate parameters of low levels of counterproductive performance. Quantile regression analysis, as an alternative proposed by Van Zyl and De Bruin (2018), has been demonstrated and can be used to explore the strength of the relationship between personality aspects and job performance across different score continuums.

The present study was the first to demonstrate the possibility of a composite *Heroic* trait, which seems to be a marker for high potential. It would be interesting to see if the predictive validity of this composite trait could be replicated for leadership effectiveness in other contexts. The present study is further confined to South African employees. However, it might be meaningful to explore the cross-cultural validity of the findings in surrounding border countries. For example, future studies could explore the measurement invariance of the 10 aspects across different nations in Southern Africa. The differential prediction, in terms of slope and regression line, could also be explored across nations in Southern Africa. Finally, the present study provides a one-shot perspective on the relationship between personality and performance. Within-person variability in performance is argued to impact the criterion validity of personality. For example, when an employee is in a transitionary period in their career, and therefore must contend with the chaos associated with change, aspects related to the meta-trait *Plasticity* might be more predictive of performance. By contrast, when a current job role needs to be maintained and a fair degree of mastery in present tasks has been attained, aspects related to the meta-trait *Stability* might ensure the requisite striving to ensure performance (Dalal et al., 2014).

## Conclusion

Few studies, with no identifiable evidence of such studies in South Africa, investigated the criterion validity of the 10 personality aspects for five narrow generic dimensions of performance. The present study highlights the importance of a more nuanced understanding of the work-related impact of personality and the associated implications for selection decisions and development suggestions. *Industriousness* and *Intellect* appear to be salient predictors of performance, perhaps because of the overrepresentation of professionals in the sample. Future studies could investigate the predictive validity of the 10 personality aspects for narrower performance dimensions in other contexts.

## Footnotes

1 The researchers of the current study did not think that occupational characteristics would impact the outcomes. No explicit hypotheses were formulated at the initiation of the study, and the potential influence of occupational characteristics was retrospectively regarded as an explanatory factor for the observed results. This is explicitly in the discussion to avoid post-hoc harking.
